# Coronary Aneurysm: An Enigma Wrapped in a Mystery

**DOI:** 10.1055/s-0039-1688467

**Published:** 2019-10-15

**Authors:** Davide Carino, Arvind Agarwal, Mrinal Singh, Judith Meadows, Bulat A. Ziganshin, John A. Elefteriades

**Affiliations:** 1 Aortic Institute, Yale-New Haven Hospital, Yale School of Medicine, Yale University, New Haven, Connecticut; 2 Department of Radiology, Yale-New Haven Hospital, Yale School of Medicine, Yale University, New Haven, Connecticut; 3 Department of Cardiovascular and Endovascular Surgery, Kazan State Medical University, Kazan, Russia

**Keywords:** coronary artery aneurysm, coronary aneurysm, aneurysm

## Abstract

Coronary aneurysms are defined as localized dilatations of the coronary arteries. In this review, we will analyze the most important aspects of this rare condition while trying to provide answers to the following questions: What is a coronary aneurysm? What causes coronary aneurysm? Do coronary aneurysms cause symptoms? Can coronary aneurysms rupture? How do we treat coronary aneurysms?

## Introduction

### Editor's Note

*John A. Elefteriades, MD*

**Fig. 1:**
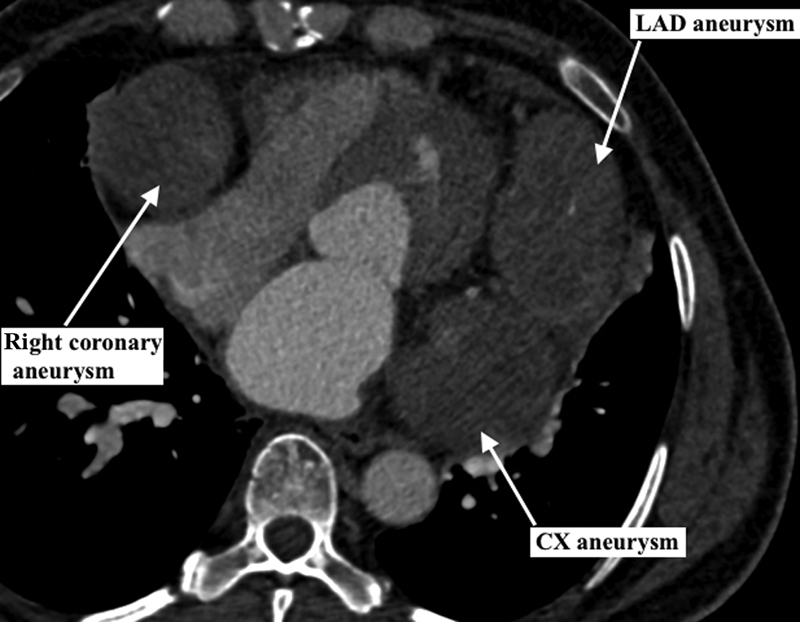
Computed tomography angiography showing giant coronary aneurysm involving the left anterior descending (LAD), the circumflex (CX) artery, and the right coronary artery. The patient is a 53-year-old woman with no cardiovascular risk factors or positive anamnesis for Kawasaki disease who presented to the emergency department for angina. Electrocardiography showed diffuse repolarization abnormality but no ST-segment elevation. Troponin levels were elevated and a diagnosis of acute coronary syndrome, non-ST-elevation myocardial infarction was established.

*When this dramatic case ( Video 1 , Fig. 1 ) was brought to our attention by Dr. Arvind Agarawal, I was not certain what therapy to recommend. Medical therapy? What could that be? Surgical treatment? What could that be? CABG, CABG with ligation? Should transplantation be offered? Is anticoagulation necessary or appropriate? What agent? As few surgeons have large experience with coronary aneurysms (especially so dramatic and widespread as in this case), I asked Dr. Davide Carino to review the data for us this report follows.*

**Video 1**

Coronary angiography followed by computed tomography angiography.

## What Is a Coronary Aneurysm?

Coronary aneurysms are localized dilatations of the coronary arteries, with a diameter 50% greater than the adjacent normal vessel. Generalized dilatation, involving greater than half of the vessel length, is termed coronary ectasia. 1 Great part of coronary aneurysms is fusiform involving the whole circumference of the vessel, but some saccular aneurysms have been described. 1 2 Prevalence ranges from 0.3 3 to 4.9% 4 of patients who undergo coronary angiography. The first case was reported by Morgagni in 1761, 5 while the first ante-mortem diagnosis was made in 1958. 6 When the maximum diameter of the artery exceeds 2 cm, the coronary aneurysm can be described as being “giant”. 7 The great majority of coronary aneurysm cases involve a single artery (or branch), predominantly the right coronary artery. 1 Involvement of the left main coronary artery is rare. 8 Coronary aneurysms (especially giant cases) are more frequently seen around the atria, suggesting focal vessel weakness in these regions.

## What Causes Coronary Aneurysm?

Etiology is variable. Causes include atherosclerosis, Kawasaki disease (KD), and vasculitic injury. Congenital aneurysms are frequently giant. 7 Iatrogenic cases have also been described following percutaneous coronary intervention (PCI). 9

Coronary aneurysms caused by KD, an immunologically mediated vasculitis, deserve particular attention for their genetic component and distinct natural history. KD is a leading cause of acquired heart disease in infants and young children. 10 The mainstay of treatment is intravenous immunoglobulin. Generally, coronary aneurysms develop in 3 to 5% of treated patients, rising to 20 to 25% in the untreated. 11

The large discrepancy in outcomes following immunomodulatory therapy has led many to investigate the genes that may be implicated in the pathogenesis of coronary aneurysms in KD. The inositol 1,4,5-triphosphate receptor type 3 ( *ITPR3* ) gene plays a critical role in the development of many autoimmune diseases, including Type 1 diabetes mellitus, systemic lupus erythematosus, rheumatoid arthritis, and Grave's disease. Moreover, the human leukocyte antigen (HLA) is linked with immune-mediated vascular diseases. HLA-B-associated transcript ( *BAT* ) genes belong to a group within the HLA class I region. Single-nucleotide polymorphisms in *ITPR3* , 12 *BAT-2* , and *BAT-3* have all been associated with the development of coronary aneurysm in patents with KD. 13 Other candidates include the protein-coding genes *NEBL* (rs16921209) and *TUBA3C* (rs17076896), whose function is associated with cardiac muscle and tubulin. 11

Familial cases of coronary aneurysm in KD patients are rarely reported. Only two instances have been described: one in a mother and her son, 14 and another in two siblings (brother and sister). 15

From a pathologic perspective, two different vasculitic processes can be identified in the coronary arteries affected by KD. One is an acute self-limiting necrotizing arteritis, 16 with neutrophilic infiltrate originating from the vessel lumen. It is associated with extensive necrosis of all layers of the vessel wall, which may result in saccular aneurysm. This process is limited to the first 2 weeks following onset of fever. The other is a subacute/chronic vasculitis that begins within the first 2 weeks but can persist for months or years. 16 It is characterized by a predominately lymphocytic inflammatory infiltrate that originates in the adventitia. Its severity is extremely broad, ranging from a mild arteritis to a highly destructive panarteritis. In saccular aneurysms secondary to the acute process, there is no trace of tunica media or internal or external elastic laminae. Conversely, these layers can be identified in aneurysms secondary to the subacute/chronic vasculitis. Furthermore, coronary aneurysms caused by the subacute/chronic vasculitis can be saccular or fusiform. 17 These different pathologic characteristics alter the natural history of these aneurysms.

Atherosclerotic coronary aneurysms are characterized by hyalinization and lipid deposition in the intima, often with focal calcification. 18 The atherosclerotic process extends to the media with degeneration of smooth muscle cells and their replacement with hyalinized collagen. 19 An association between atherosclerotic coronary aneurysm and abdominal aortic aneurysm has been reported. 18 20 21 It is postulated that coronary aneurysms result from excessive pressure produced by alternating high/low blood flow in a vessel weakened by atherosclerosis. 18 22 Atherosclerotic aneurysm can be either pre- and poststenotic. 22

Finally, congenital coronary aneurysms are histologically similar to atherosclerotic aneurysms, but without lipid deposition. 23

## Do Coronary Aneurysms Cause Symptoms?

Although coronary aneurysm are intrinsically asymptomatic, they can cause angina and myocardial infarction secondary to vasospasm or intraluminal thrombosis, with vessel occlusion or distal embolization. 18 24 25 Congenital giant coronary aneurysms have been associated with fistula formation to the ventricles, 7 26 leading to congestive heart failure.

Previously, it was believed that disruption of smooth muscle cells precludes significant vasoconstriction of the coronary aneurysm. However, ischemic electrocardiographic changes have been seen following administration of the vasoconstrictor ergonovine maleate. 27 Coronary aneurysm spasm during PCI has also been described. 28 These data illustrate that non-KD-related coronary aneurysm are capable of vasocontraction despite an attenuated tunica media.

## Can Coronary Aneurysms Rupture?

The risk of rupture of coronary aneurysms is directly correlated with their etiology. Ruptures have been reported in young patients with KD during the acute phase of the diseases. 16 As mentioned, during this period, a necrotizing arteritis causes extensive necrosis of all vessel layers, thereby predisposing to rupture. However, only a single case of KD-related coronary aneurysm rupture has been reported beyond the acute phase of the disease. 14

Rupture of atherosclerotic aneurysm is a very rare and unpredictable event. 1 4 29 30 Only two cases are reported, and the aneurysm diameter exceeded 8 cm in both cases. 31 32

The ability to induce vasospasm, combined with the extremely low risk of rupture, make the clinical behavior of atherosclerotic coronary aneurysms distinct from the histologically similar aneurysms of the descending thoracic and abdominal aorta.

The explanation for this low risk of rupture has not been elucidated. We speculate that absence of systolic flow in the coronary circulation with the subsequent lack of hypertensive peak can explain this peculiar behavior.

## How Do We Treat Coronary Aneurysms?

The rarity of coronary aneurysms makes it difficult to standardize treatment or firmly establish guidelines supporting optimum management. In general, patients with small asymptomatic coronary aneurysms without significant occlusive atherosclerotic coronary disease do not require any treatment. Aneurysms with symptoms secondary to intraluminal thrombosis can be managed with anticoagulation therapy or surgery. To date, there are no data comparing medical management with surgical treatment. However, most authors agree that surgical treatment would be indicated based more on the severity of the underlying coronary disease rather than the presence of the aneurysm itself.

Surgery consists of ligation of the aneurysm followed by coronary artery bypass grafting (CABG). Matsubayashi et al report a novel surgical approach in a case involving the distal left main coronary artery only. 8 They performed a direct anastomosis of the circumflex artery to the proximal left main and sutured a 10-mm Dacron interposition graft between the dilated ostium of the left anterior descending artery and the proximal aorta. Surgical treatment for giant aneurysm involving two vessels has been reported as well. 33 34 Finally, endovascular treatment of saccular aneurysm with coil embolization to prevent rupture has been reported. 35

For giant coronary aneurysms associated with fistula, surgery is mandatory to prevent development of end-stage heart failure. 7 In these cases, the aneurysm is opened and the fistulous ostia are closed by direct suture or with a pericardial patch. Then, if aneurysm dimensions and vessel quality allow, the artery is reconstructed. Otherwise, the aneurysm is ligated proximally and distally, and CABG is performed.

## Conclusions

Coronary aneurysm is a rare condition of variable etiology, with a strong genetic component in cases attributable to KD. Coronary aneurysms caused by KD manifest histologic peculiarity that carries a greater risk of rupture. Atherosclerotic aneurysms, although similar to descending thoracic and abdominal aortic aneurysm histologically, are clinically different. They exhibit inducible vasospasm and the risk of rupture is exceedingly low, perhaps due to the absence of hypertensive peak. Given the rarity of arteriosclerotic coronary aneurysms, the particular clinical scenario must guide the choice of management. Options include medical therapy with anticoagulation and surgery with ligation of the aneurysm and CABG. Coronary aneurysm with fistula mandates surgery to prevent end-stage heart failure. In our case ( Video 1 , Fig. 1 ), three giant coronary aneurysms involved all the main coronary arteries. Heart transplantation may be considered for such cases.
